# Food Industry Donations to Academic Programs: A Cross-Sectional Examination of the Extent of Publicly Available Data

**DOI:** 10.3390/ijerph17051624

**Published:** 2020-03-03

**Authors:** Marie A. Bragg, Brian Elbel, Marion Nestle

**Affiliations:** 1 Department of Population Health, New York University School of Medicine, New York, NY 10016, USA; Brian.Elbel@nyulangone.org; 2 Department of Nutrition, New York University School of Global Public Health, New York, NY 10012, USA; 3 New York University Wagner School of Public Policy, New York, NY 10021, USA; 4 Department of Nutrition and Food Studies, New York University Steinhardt School of Culture, Education, and Human Development, New York, NY 10003, USA; marion.nestle@nyu.edu

**Keywords:** food industry, academic donations, conflicts of interest

## Abstract

No studies have documented the prevalence of the food industry’s funding of academic programs, which is problematic because such funding can create conflicts of interest in research and clinical practice. We aimed to quantify the publicly available information on the food industry’s donations to academic programs by documenting the amount of donations given over time, categorizing the types of academic programs that receive food industry donations, cataloguing the source of the donation information, and identifying any stated reasons for donations. Researchers cataloged online data from publicly available sources (e.g., official press releases, news articles, tax documents) on the food industry’s donations to academic programs from 2000 to 2016. Companies included 26 food and beverage corporations from the 2016 Fortune 500 list in the United States. Researchers recorded the: (1) monetary value of the donations; (2) years the donations were distributed; (3) the name and type of recipient; (4) source of donation information; and (5) reasons for donations. Adjusting for inflation, we identified $366 million in food industry donations (N = 3274) to academic programs. Universities received 45.2% (*n* = 1480) of donations but accounted for 67.9% of total dollars given in the sample. Community colleges, schools (i.e., preschool, elementary, middle, and high schools), and academic nonprofits, institutes, foundations, and research hospitals collectively received 54.8% of the donations, but made up less than one-third of the monetary value of donations. Half of the donations (49.0%) did not include a stated reason for the donation. In our sample, donations grew from $3 million in 2000 to $24 million in 2016. Food companies in our sample donated millions of dollars to universities and other academic programs but disclosed little information on the purpose of the donations. Achieving transparency in donation practices may only be possible if federal policies begin to require disclosures or if companies voluntarily disclose information.

## 1. Introduction

Corporations have a long history of providing financial support to the academic sector in the United States. Such financial support can take the form of student scholarships [1], research endowments [2], and other charitable gifts that supplement the operating costs of schools, universities, and academic research hospitals [3]. Although the recipients of industry donations benefit from these contributions, the lack of transparency regarding industry donations to academic programs has recently received considerable scrutiny [3,4]. One concern is that industries marketing harmful products have a vested interest in maintaining relationships with academic institutions that may positively portray industry products or minimize evidence for harmful effects.

Tobacco, pharmaceutical, and chemical industries, for example, have funded dozens of research studies that show outcomes favorable to their products [5,6]. Industry funders can also directly influence scientific design and analyses, which may introduce biases that favor industry products. One study showed that industry-funded trials of vaccines, drugs, and medical devices involved the industry in data analyses for 73% (*n* = 146) of the trials [7]. Although industry-funded studies may not always produce biased results, partnerships between industry and academia can also create opportunities for companies to distribute samples of their products, brand teaching materials [8,9], access students through scholarships [10], participate in conferences and seminars, or in other ways influence the content and direction of academic reports [8,9]. These factors have been shown to influence physicians’ prescription practices in ways that may occur unconsciously and be difficult for recipients to recognize [11].

Although much less is known about academic funding distributed by food and beverage companies, restaurants, supermarkets, and agricultural corporations, (collectively the food industry), food industry-sponsored research often supports industry objectives [8,12,13,14,15,16,17,18,19], and industry relationships with academia have influenced medical journalism [19] and public policy [3]. In 2011, for example, the American Beverage Association donated $10 million for childhood obesity prevention initiatives to the Children’s Hospital of Philadelphia when the City Council was considering a soda tax proposal [20]. The Council rejected the tax, eliciting further concern about the industry’s influence on academic programs and public health policies. One study found that between 2011 and 2015, the beverage industry contributed to 96 national health-affiliated groups, and 63 of them focused on public health [21]. Public health experts have expressed concern about food industry donations to health-affiliated groups because corporations are inextricably bound to the obligation to sell products, some of which are at odds with good health and the individuals’ ability to maintain a healthy weight [8,22]. 

Increasing the transparency of the food industry’s relationships with academic groups is critical for preventing and managing problematic conflicts of interest. One of the most compelling examples of the value of transparency involved journalists reporting financial data that ultimately led to Coca-Cola’s disclosure of donations to health-related programs. In August 2015, the New York Times reported that Coca-Cola was funding a research group called the Global Energy Balance Network [23]. For several months, Coca-Cola was publicly criticized by public health experts who said the Network’s mission was to shape obesity research and minimize criticism of the role of sugary drinks in health [23]. In November 2015, the Network’s research group at the University of Colorado returned $1 million to Coca-Cola, and by December 2015, Coca-Cola dissolved the Network. Coca-Cola also began posting the details of its donations to public health research and health programs on its website amidst increasing pressure to improve the transparency of its donations [3,24].

The benefits of transparency are not limited to the food industry. The truth^®^ campaign designed its anti-tobacco advertising to disclose the manipulative behaviors of the tobacco industry; its adverts were credited with achieving significant decreases in smoking among youth in the United States [25]. The World Health Organization’s (WHO) 2008 report on the Tobacco Industry’s Interference with Tobacco Control also reinforces the need for transparency and monitoring of industry behavior: “WHO is well aware of the long history and the extent of tobacco industry efforts to avoid, delay, and dilute the advancement of effective tobacco control policies and interventions. The position of WHO is that it will not accept funding from the tobacco industry. Understanding and effectively counteracting efforts by the tobacco industry and its allies to oppose tobacco control are crucial. Given this reality, the WHO Tobacco Free Initiative monitors and draws global attention to the activities and practices of the tobacco industry [26].” Given the food industry’s history in delaying and diluting the advancement of public policies and research on obesity, there is an urgent need to increase the transparency of food industry donations to academic programs. Such transparency may help prevent conflicts of interest in research and clinical practices and increase awareness of how donations may buy goodwill.

Despite increasing attention to the need for transparency regarding partnerships between the food industry and academic programs [3], studies on food companies’ contributions to academia have largely been limited to case studies [19], those with small samples [13,27], or commentaries [8,9,22,28,29]. These studies provide valuable insights on the food industry’s donation practices, but there are currently four gaps in this literature. First, the lack of large datasets on food industry donations to academic programs means researchers cannot begin to understand whether this is a rare occurrence involving a few companies, or a more widespread phenomenon that warrants greater concern. Second, if the literature only provides small amounts of data, then this suggests we currently lack comprehensive data on which academic sectors (e.g., universities, research hospitals, academic nonprofits) are most at risk of conflicts of interest. Third, most published case studies and commentaries have not systematically searched for academic donations, which limits our ability to compare sources of data that include a large group of companies. Finally, no studies have examined the companies’ stated reasons for donations, which limits our ability to monitor and address potential conflicts of interest in research or clinical practices. Addressing these gaps would equip researchers, journalists, and academic sectors with critical information for preventing conflicts of interest and advocating for increased transparency from companies.

This study aims to address these four gaps in the literature by: (1) quantifying publicly available information on the food industry’s donations to academic programs by documenting the amount of donations given over time; (2) categorizing the types of academic programs that receive food industry donations; (3) cataloguing the source of the donation information; and (4) identifying any stated reasons for donations.

## 2. Materials and Methods

We conducted a cross-sectional, observational evaluation of food industry donations given to academic programs between 2000 and 2016. To select companies for our sample, research assistants identified all 26 companies associated with the food industry that were ranked in the Fortune 500 list for 2016 [30]. The Fortune 500 is an annual list published by Fortune magazine that ranks the 500 largest corporations in the United States according to total revenue. We operationalized the food industry as any company whose primary objective is to sell food or beverage products (e.g., Coca-Cola; supermarkets) or support the production of food through agriculture (e.g., Monsanto; Cenex Harvest States [CHS]). 

**Figure 1:**
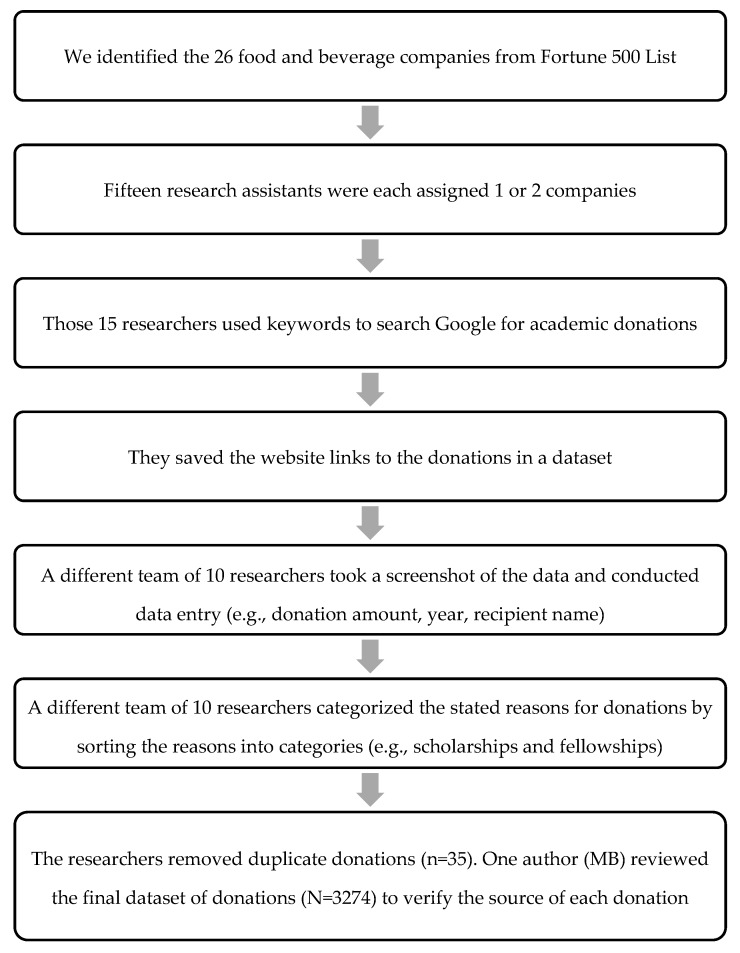
Flow chart describing the online search processes and data entry, coding, and cleaning.

During 2016, we randomly assigned one or two of the 26 companies to 15 research assistants, assigning no single company to more than one assistant. We trained the research assistants to search for donations from their assigned company to an academic program between 2000 and 2016 using the procedures described in Figure 1. Specifically, we defined academic programs as those that described their primary mission as education (e.g., universities, schools), academic research, or that directly supported education or research through a foundation or non-profit. Because our study focused on conflicts of interest in the academic sector, we excluded hospitals that were unaffiliated with universities and institutes, nonprofits, and foundations that focused on advocacy as their primary mission, even if they supported research (e.g., Susan B. Komen Foundation). We selected 2000 as the year for the start of the data collection period because widespread Internet use began in the early 2000s [31], which increased our likelihood of finding donation information online.

We instructed research assistants to use www.Google.com to identify donations by using every combination of the following keywords: name of the assigned food or beverage company plus the words “donation”, “gift”, “contribution”, “funding”, “grant”, “financial support”, “academic”, or “scholarship”. These searches yielded results that included academic (e.g., university, hospital, school) websites, food company websites, media press releases, tax documents, and other sites unrelated to academic donations. Research assistants clicked each link identified during the search to determine if the site included donation information. The first three research assistants were instructed to record the time point at which they could no longer identify new donations (i.e., when more than one hour passed without finding a new donation). Because this time point occurred at roughly five hours for each of the three assistants, we instructed the remaining 12 assistants to limit their search to five hours unless they continued to find additional donations.

Collectively, the research assistants searched online for food industry donation information for 130 hours. We asked them to include information from official company websites, press releases, and reputable media sources (e.g., The New York Times, The Wall Street Journal, and The Washington Post). One of the authors (MB) trained each research assistant to identify reputable news sources as national news outlets with the most online visitors per month, according to Nielsen and comScore [32]. We also included news sources from less well-known sources (i.e., small, local newspaper) if their articles linked to websites that confirmed the donation or contained direct quotes from donors or recipients that confirmed the donation. To maximize each research assistant’s ability to find donations during their five-hour search, we asked them to enter the link to the donation information into our dataset without recording specific donation information. A separate team of 10 research assistants visited the website links gathered by the first set of researchers and collectively spent 500 hours recording and organizing data on the: (1) monetary value of the donations; (2) year the donation was distributed; (3) name and type of recipient; (4) source of donation information; and (5) reason for the donation. Assistants also took a screenshot of the website in case it was removed at a later date.

In order to categorize the type of recipient, research assistants used the keywords that described the recipient on its home webpage. They sorted recipients into seven categories that related to any type of academic program including university; community college; preschool, elementary, middle, or high school; academic nonprofit; academic institute; academic foundation; and research hospital. We chose to include several of these categories because they were used in other studies (i.e., university [7], academic hospital [11], and institute [8]), but we added the other categories during the data collection period to provide more granular detail about the types of recipients. Research assistants also labeled the source of the documentation: (1) annual report from donor website; (2) annual report from recipient website; (3) tax document (i.e., any forms used in the United States for taxpayers and tax-exempt organizations to report financial information to the government); (4) news articles; (5) press release featured on donor website; or (6) press release featured on the recipient website. Research assistants also reviewed the screen shots for any information that described the donation’s purpose and sorted them into one or more of the following ten categories: matching gifts (i.e., a corporate donation that matches employee donations to a nonprofit organization); educational initiatives; general operations support (e.g., technological equipment); scholarships and fellowships; health and human services (e.g., obesity prevention programs); support for communities of color; research; endowed chair or professorship; and miscellaneous programs (e.g., career counseling programs).

After the dataset was complete, we searched for and removed duplicate donations (*n* = 35) that we identified on a tax document and a different data source. We also adjusted the donation amounts for inflation by calibrating them to the year 2016, because this was the final year of our data collection period. Then, we quantified the total number and amount of the donations for the entire sample and for each company. Researchers also calculated the frequency of the stated reasons for donations and determined the percentage of the sample that was identified via tax documents, news articles, food industry websites, or donation recipient websites. We logged trends in the number of donations and donation amounts given over time across the entire sample.

## 3. Results

### 3.1. Increase in the Number and Monetary Value of Donations over Time

**Figure 2:**
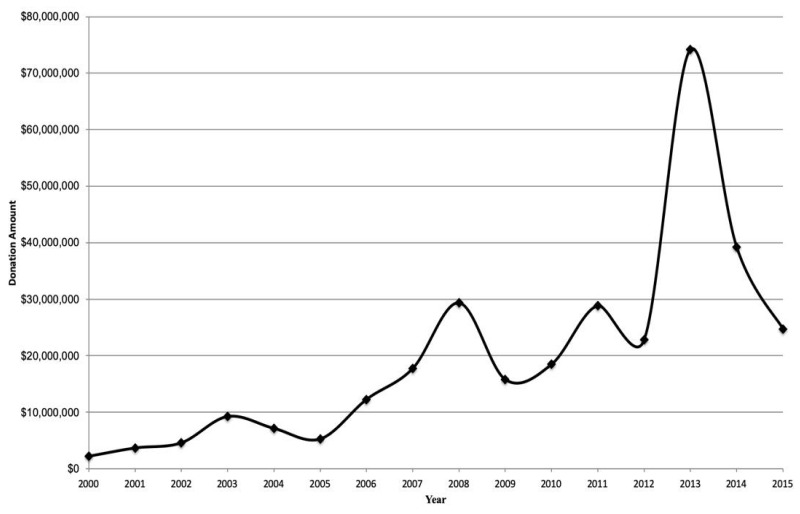
Trends in donations made to academic institutions by food and beverage companies from 2000 to 2015.

Figure 2 shows an overall increase in donations from 2000 to 2015. We excluded 2016 data from the figure because tax documents were not yet available for 2016 at the time of data collection. The figure shows that between 2000 and 2013, average donation amounts increased from $3 million (*n* = 48 donations) to $74 million (*n* = 300 donations). Then from 2014 to 2015, the number and monetary value of donations in our sample declined, totaling $25 million in 2015. We identified $24 million in 2016, and the decline during the latter years may reflect that tax information was not yet fully available at the time of data collection. 

### 3.2. Monetary Value of the Donations and Years the Donations Were Distributed

Using R Version 3.5, we compiled and analyzed data from 3274 donations by the 26 food and beverage companies from 2000 to 2016. Table 1 lists the donors, amounts, and number of donations, and the number of years for which we found donations for a given company. For the 26 companies, the amounts totaled $366 million, adjusting for inflation. The Hormel Food Corporation appeared to be the largest donor; its 61 donations came to more than $108 million and accounted for 29.5% of donations we identified in terms of dollar value. 

Table 2 lists the 25 largest individual donations to academic programs in our sample. In 2016, Tyson Foods Corporation gave the largest individual donation ($15 million) to Arkansas Children’s Northwest, a children’s hospital affiliated with the University of Arkansas for Medical Sciences. The press release stated that the donation was earmarked for construction of the children’s hospital including a clinical area that would be labeled with the company’s name. Monsanto gave the second largest individual donation ($13,789,983) to Texas A&M University Texas AgriLife Research in 2013. The press release reported Monsanto provided the donation to fund an academic fellowship program to “support the next generation of scientific leaders working to improve rice and wheat breeding.”

### 3.3. Types of Recipients

Forty-five percent (45.2%; *n* = 1482) of donations were given to universities; 35.5% (*n* = 1164) were given to preschool, elementary, middle, and high schools; and the remaining donations were to academic nonprofits (7.9%; *n* = 269), foundations (7.9%; *n* = 259), hospitals (1.3%; *n* = 44), institutes (1.1%; *n* = 36), or community colleges (0.6%; *n* = 20) (Table 3). The two largest categories of recipient types (i.e., universities and schools) received more donations (*n* = 920) from Publix Supermarkets than from any other food or beverage company. Darden Restaurants accounted for the second highest number of donations to universities and schools (*n* = 412).

### 3.4. Source of Donation Information

Using our search strategy, we found 491 documents and 79% (*n* = 388) of them were tax documents spanning from 2000 to 2016. The remaining documents included news articles (5.3%; *n* = 29), annual reports on recipients’ websites (4.5%; *n* = 26) or on donors’ websites (3.5%; *n* = 22), and press releases featured on donors’ websites (3.5%; *n* = 17) or on recipients’ websites (1.8%; *n* = 9).

### 3.5. Stated Reasons for the Donations

Research assistants were unable to identify a reason for half (49.0%; *n* = 1607) of the academic donations. Among the 1667 remaining donations, the identified reasons represented nine broad categories that were not mutually exclusive (i.e., some donations listed more than one reason). The largest categories included “matching gifts” without a description of the gift being matched (36.8%; *n* = 615, median amount = $522.50); educational initiatives (25.2%; *n* = 420, median amount = $11,190); general operations support (19.0%; *n* = 316, median amount = $1493); and scholarships and fellowships (9.2%; *n* = 154, median amount = $67,844).

## 4. Discussion

To our knowledge, we generated the largest food industry donation database to date. The total monetary value of donations in our sample exceeded $366 million. Universities received less than half (45.2%) of the donations in the sample but accounted for more than two-thirds of the total monetary value of the donations. In contrast, community colleges, schools, and academic nonprofits, institutes, foundations, and hospitals collectively received 54.8% of the donations, but made up less than one-third of the monetary value of donations in the sample. It is possible that the imbalance in the types of academic programs that received food industry funding reflects the possibility that universities publicize donations more than other academic groups. However, we identified the majority (79.0%) of our donation data from tax documents, making it unlikely that universities merely publicize donations more than other groups. 

Our data also provide new examples of potential conflicts of interest involving a variety of companies and universities. Among the 25 largest donations in our sample, four donations appeared to have conflicts of interest in research or education. Coca-Cola, for example, donated more than $4 million to Georgetown University for “…the inaugural global human development chair holder.” Monsanto provided support for “fellowships to support the next generation of scientific leaders working to promote rice and wheat breeding” and CHS—a global agribusiness—provided a donation “…intended to transform agriculture education from kindergarten to higher education.” Pepsi provided more than $6 million in grant funding “…in 2008 [to] found the Columbia Water Center.” The website for the Columbia Water Center at Columbia University discloses that PepsiCo was a previous supporter, but we were not able to identify any documents on the website that disclose Pepsi’s level of involvement in the Center’s research. These four donations reinforce concerns raised by public health experts regarding the food industry’s involvement with health-related science, policy, and education [8,9,27].

Several patterns in our data suggest that relationships with universities are highly valued by the food industry. Universities received 17 of the 25 largest donations in our sample (Table 2), suggesting the food industry maintains a particularly strong interest in this academic sector. Another indicator of the food industry’s interest in universities is the comparatively low value of donations given to schools and other academic nonprofits. Of the 10 companies who donated the highest dollar amounts in our sample (Table 1), four companies collectively gave more than $83 million with just 22 donations. The other six companies, however, collectively gave more than $221 million with a total of 2015 donations. The majority of those 22 donations were given to universities, whereas many of the 2015 donations were provided to schools. It is possible that this pattern reflects the food companies’ interest in universities. It is also possible, however, that larger donations are required to generate interest from universities, whereas schools, scholarship foundations, or other academic nonprofits may be more willing to accept small donations. 

Our findings also provide new insights into the types of donations made by food and beverage companies. Three of the largest categories of earmarked donations included educational initiatives (25.1%; *n* = 420); general operations support (18.9%; *n* = 316); and scholarships and fellowships (9.2%; *n* = 154). Although such support may enable academic programs to provide valuable educational initiatives and increase student access to education, it is also possible that these relationships might generate long-term goodwill toward companies in ways that create conflicts of interest. Very few donations in our sample (*n* = 304) were earmarked for scholarships, endowed university chair positions, research, supporting communities of color, and health and human services projects (e.g., maternal health interventions). Despite the small number of collective donations in these categories, some of these donations were among the largest in the sample (e.g., university-based research grants; Dr. Pepper’s $9 million tuition giveaway in 2008).

Half of the donations we identified (49.0%) did not include a stated reason for the donations, and it is impossible for us to discern why certain gifts were not earmarked for a specific purpose. This lack of disclosure demonstrates the need for policies that require increased transparency for donation practices. Universities, academic institutes, and hospitals may be particularly vulnerable to conflicts of interest because they generate and disseminate health research or conduct clinical work with patients. These types of academic programs should create a uniform system for disclosing industry donations to reduce the risk of conflicts of interest affecting research and clinical practices. 

One example of a donation disclosure tool that could be used by academic programs is the Kaiser Family Foundation database called “Pre$cription for Power”, which catalogued 12,000 donations made by pharmaceutical companies to patient advocacy groups [33]. Patient advocacy groups wield tremendous political power in the United States, and companies value being associated with these groups because of their political influence. “Pre$cription for Power” found that 14 companies donated $116 million to patient advocacy groups during 2015. But those companies reported spending just $65 million on political lobbying in the same year. Because companies are not required to disclose donations made to these groups in the same way that they must disclose political lobbying expenditures, companies can minimize the appearance of lobbying via relationships with patient advocacy groups. Academic programs, therefore, should increase the transparency of their relationships with corporations by entering the donation information into a database modeled after “Pre$cription for Power”. 

Despite our inability to discern how companies decide how much to donate and which organizations should receive gifts, these donations likely generate a range of benefits for the companies. Research studies on corporate social responsibility demonstrate that consumers and employees feel goodwill and higher loyalty toward companies who engage in activities that support social and environmental causes [34,35,36]. One cross-sectional study of 103 children ages 10–14 years found that children perceived food companies as “kind” and “cool” when they supported sporting events [37].

The strengths of our study include the large number of companies in our sample and our extensive data collection methods, which enabled us to generate new insights on the types of academic programs that receive donations (e.g., preschools, universities) and create a comprehensive catalogue of reasons for donations among the 1667 donations that were earmarked for a specific purpose. In contrast, most previous studies on food companies’ contributions to academia have largely been limited to case studies [19], studies with small samples [13,27], commentaries [8,9,22,28,29], or summaries of academic institutions’ recommendations regarding the management of conflicts of interest [38]. 

This study has several limitations. Our search missed donations that were not highly publicized, those on websites that had deleted older information or not disclosed such information, or donations from shell foundations and companies. Another limitation of our study is that the data on the growth in donations over time may be attributable to secular trends in Internet use, recent public pressure to increase transparency in industry donation practices, or removal of older donation information from websites. Finally, it is possible that our Google search terms produced biased search results that may have led us to miss relevant websites. For example, we used search terms such as “[company name] [synonyms for donation]” but not “[company name] endowed chair” or “[company name] hospital”, meaning our search procedures likely produced an incomplete list of the food industry’s relationships with academic programs. 

Gaps in our data point to unanswered research questions that could be addressed by future research. Given we did not capture the full scope of food industry donations to academic programs, future studies could prospectively track online donation announcements to reduce the likelihood of missing donation information that was deleted or replaced with new web content. Any donations that did not receive online publicity, however, would be missed. Future studies could also examine academic donations made by companies excluded from the Fortune 500 list such as Mars or Associated British Foods. Including a wide range of international brands in future studies could highlight similarities and differences in donation practices across different countries, which would guide public policies that aim to increase transparency. Studies could also examine other companies that have some role in the production or sale of food and beverage products (e.g., Walmart, DuPont).

## 5. Conclusions

In sum, our findings reinforce the need for the food industry to provide more thorough details regarding their donation practices. The implications of our study include increasing awareness of the food industry’s donation practices, which may generate more pressure for companies to disclose donations through voluntary initiatives or public policies. If more companies created donation disclosure websites similar to Coca-Cola’s Transparency website, researchers, advocates, and policymakers could better monitor and address potential conflicts of interest that may arise from donations to academic programs.

## Figures and Tables

**Table 1:** Summary of public information on 26 food company donations to academic programs between 2000 and 2016.

Name of Donor Company	Total Donation Amount, Adjusted for Inflation	Median Donation Value Per Year, Adjusted for Inflation	Total Number of Donations	Number of Years Actively Donating	Median Number of Donations Per Year
Hormel Foods Corporation	$108,104,640	$441,907	61	15	4
The Coca-Cola Company	$32,422,036	$1,651,500	62	7	2.5
PepsiCo, Inc.	$31,267,550	$1,567,500	6	5	1
Kellogg Company	$27,350,211	$365,040	63	9	3
Tyson Foods, Inc.	$22,284,244	$4,474,950	5	2	2.5
Darden Restaurants, Inc.	$20,926,408	$2220	517	14	32.5
Publix Supermarkets, Inc.	$16,741,967	$550	1016	15	46
Monsanto Company	$16,725,887	$822,822	6	4	1.5
ConAgra Brands Inc.	$15,460,877	$1672	296	13	17
Land O’Lakes, Inc.	$13,540,449	$304,200	5	1	5
CHS Inc.	$12,743,371	$191,925	37	4	7.5
Yum! Brands, Inc. ^a^	$11,840,590	$66,692	24	9	1
General Mills, Inc.	$11,761,610	$16,000	178	15	6
Dr Pepper Snapple Group	$8,920,000	$8,920,000	1	1	1
The Kraft-Heinz Company ^b^	$3,415,716	$16,815	154	13	2
The Hershey Company	$2,181,063	$10,450	76	15	5
Dean Foods	$2,013,811	$25,897	38	6	6
Starbucks Corporation	$1,838,488	$12,100	164	10	14.5
The Kroger Company	$1,639,915	$5137	130	4	22
Archer Daniels Midland Co.	$1,567,500	$1,567,500	1	1	1
J.M. Smucker Company	$1,560,000	$780,000	2	2	1
McDonald’s	$615,965	$157,982	3	2	1.5
Campbell’s Soup Company	$609,814	$528	397	8	53
United Natural Foods, Inc.	$119,128	$18,496	5	2	2.5
Nestle USA, Inc.	$44,599	$44,599	1	1	1
Mondelez Intl, Inc.	$30,911	$10,770	4	2	2
Total	$366,475,333	$48,900	3274	$\tilde{x}$: 5.5	$\tilde{x}$: 2.5

^a^ Includes Kentucky Fried Chicken, Pizza Hut, and Taco Bell, which are companies owned by Yum! Brands, Inc. ^b^ Includes Oscar Meyer, which is owned by The Kraft Heinz Company.

**Table 2:** Twenty-five largest academic donations by food companies between 2000 and 2016, ranked by total monetary amount.

Donor Company	Name of Recipient	Year	Donation Amount ^a^	Reason for Donation	Language from Data Source
Tyson Foods, Inc.	Arkansas Children’s Northwest	2016	$15,000,000	General Operations Support	“$15 million for the construction of Arkansas Children’s Northwest”
Monsanto Company	Texas A&M University AgriLife	2013	$13,789,983	Scholarships and Fellowships	“Fellowships to Support the Next Generation of Scientific Leaders Working to Promote Rice and Wheat Breeding”
Hormel Foods Corporation	University of Minnesota	2012	$13,469,021 ^b^	General Operations Support	“Purpose of grant or assistance—Support of Operations”
Land O’Lakes, Inc.	University of Minnesota	2015	$13,050,787	Educational Initiatives	“…partnership to drive educational excellence and student development programs....”
Dr Pepper Snapple Group	Dr. Pepper Tuition Giveaway	2008	$8,979,192	Scholarships and Fellowships	“…$8 million in tuition to hard-working college students through the Dr Pepper Tuition Giveaway”
Kellogg Company	Academy for Ed Development	2011	$7,926,354	Supporting Communities of Color	“…promote family and community leadership of southern African youth as agents”
PepsiCo	Columbia University	2008	$6,688,443	Miscellaneous Program Support	“…$6 million grant in 2008 helped found the Columbia Water Center”
Tyson Foods, Inc.	University of Arkansas	2015	$5,666,755	··	··
Hormel Foods Corporation	University of Minnesota	2014	$5,738,241	General Operations Support	“Purpose of grant or assistance—Support of Operations”
Hormel Foods Corporation	University of Minnesota	2013	$5,159,969	General Operations Support	“Purpose of grant or assistance—Support of Operations”
Hormel Foods Corporation	University of Minnesota	2013	$4,812,771	··	··
Hormel Foods Corporation	Austin Public Schools	2014	$4,595,511	Educational programs	“Purpose of grant or assistance—Educational Programs”
Hormel Foods Corporation	University of Minnesota	2011	$4,376,675	General Operations Support	“Purpose of grant or assistance—Support of Operations”
The Coca-Cola Company	Georgetown University	2014	$4,055,268	Endowed Chair or Professorship	“…chosen as the inaugural global human development chair holder, supported by a $4 million gift…”
Hormel Foods Corporation	University of Minnesota	2008	$4,167,956	General Operations Support	“Purpose of grant or assistance—Support of Operations”
The Coca-Cola Company	Spelman College	2011	$3,734,454 ^b^	Scholarships and Fellowships	“…scholarships for hundreds of students, Women of Color Conference”
CHS	University of Minnesota	2016	$3,440,000	Scholarships and Fellowships	“…intended to transform agriculture education from kindergarten to higher education.”
Hormel Foods	University of Minnesota	2010	$3,416,413	General Operations Support	“Purpose of grant or assistance—Support of Operations”
Hormel Foods Corporation	University of Minnesota	2007	$3,369,036	Scholarships and Fellowships	“Purpose of payment to affiliate—Scientific Research”
Hormel Foods Corporation	Riverland Community College	2013	$3,264,937	··	··
Kellogg Company	Louisiana Public Health Institute	2011	$3,227,225	Health and Human Services	“…strengthen community-based access to physical and mental health services”
The Coca-Cola Company	Emory University	2008	$3,344,221	Scholarships and Fellowships	“to provide scholarships, fellowships, and support for sustainability projects”
Hormel Foods Corporation	Riverland Community College	2013	$3,047,230	Health and Human services	“Purpose of grant or assistance—Scholarships”
Taco Bell	Get Schooled Foundation	2015	$3,002,481	··	··
Hormel Foods Corporations	University of Minnesota	2009	$2,890,717	General Operations Support	“Support of Operations”
Total	··	··	$133,010,165	··	··

^a^ We adjusted for inflation by calibrating the donation amounts to 2016. ^b^ This donation included one or more years outside of our data collection period, meaning the actual donation was larger than the figure reported in this cell. The figure in this cell represents the estimated value of the donations given during our data collection period.

**Table 3:** List of reasons for the donations among the 1667 donations that provided at least one reason.

Reason for Donation	Number (%) of Donations with That Reason Listed
Matching Gifts	615 (36.8)
Educational Initiatives	420 (25.1)
General Operations Support	316 (18.9)
Scholarships and Fellowships	154 (9.2)
Miscellaneous Programs	124 (7.4)
Health and Human Services	57 (3.4)
Supporting Communities of Color	47 (2.8)
Research	35 (2.0)
Endowed Chair or Professorship	11 (0.7)
Total Number of Reasons Listed ^a^	1779

^a^ There were 1667 donations with one *or* two specific reasons for the gift. As some donations had more than one reason listed, this total is 1779.
